# Progress Achieved, Landmarks, and Future Concerns in Biomedical and Health Informatics

**DOI:** 10.3390/healthcare12202041

**Published:** 2024-10-15

**Authors:** Ivana Ognjanović, Emmanouil Zoulias, John Mantas

**Affiliations:** 1 Faculty for Information Systems and Technologies, University of Donja Gorica, 81000 Podgorica, Montenegro; 2 European Federation for Medical Informatics, CH-1052 Le Mont-sur-Lausanne, Switzerland; 3 Health Informatics Lab, Department of Nursing, National and Kapodistrian University of Athens, 11527 Athens, Greece; ezoulias@nurs.uoa.gr (E.Z.); jmantas@nurs.uoa.gr (J.M.)

**Keywords:** biomedical informatics, health informatics, medical informatics, education, accreditation, nursing informatics, health informatics data, ethics, security, digital health innovations

## Abstract

Background: The biomedical and health informatics (BMHI) fields have been advancing rapidly, a trend particularly emphasised during the recent COVID-19 pandemic, introducing innovations in BMHI. Over nearly 50 years since its establishment as a scientific discipline, BMHI has encountered several challenges, such as mishaps, delays, failures, and moments of enthusiastic expectations and notable successes. This paper focuses on reviewing the progress made in the BMHI discipline, evaluating key milestones, and discussing future challenges. Methods: To, Structured, step-by-step qualitative methodology was developed and applied, centred on gathering expert opinions and analysing trends from the literature to provide a comprehensive assessment. Experts and pioneers in the BMHI field were assigned thematic tasks based on the research question, providing critical inputs for the thematic analysis. This led to the identification of five key dimensions used to present the findings in the paper: informatics in biomedicine and healthcare, health data in Informatics, nurses in informatics, education and accreditation in health informatics, and ethical, legal, social, and security issues. Results: Each dimension is examined through recently emerging innovations, linking them directly to the future of healthcare, like the role of artificial intelligence, innovative digital health tools, the expansion of telemedicine, and the use of mobile health apps and wearable devices. The new approach of BMHI covers newly introduced clinical needs and approaches like patient-centric, remote monitoring, and precision medicine clinical approaches. Conclusions: These insights offer clear recommendations for improving education and developing experts to advance future innovations. Notably, this narrative review presents a body of knowledge essential for a deep understanding of the BMHI field from a human-centric perspective and, as such, could serve as a reference point for prospective analysis and innovation development.

## 1. Introduction

Health informatics is recognised as a discipline “concerned with the systematic organisation, representation, and analysis of data, information and knowledge in biomedicine and healthcare” with the “aim to contribute to high-quality, efficient healthcare and quality of life on the one hand and to progress in science on the other” [1,2]. When we look at the period of the last 50 years as a period in which health informatics, or BMHI, was built as a scientific discipline, we can see the nonlinearity trend in the development and the close connection with other disciplines and social contexts. The recent peak in effect caused by the COVID-19 pandemic has further emphasised the permanent need for improving the application of modern technologies in health and healthcare, with an additional critical analysis of the sporadic impacts and effects of application as a key challenge for further development.

To thoroughly examine and critically assess the timeline of health informatics development and analyse its achievements and challenges from various perspectives, we designed and implemented a carefully chosen qualitative methodology for a narrative review of the BMHI field. The central elements of the method involve gathering expert opinions from pioneers in the field, followed by a literature review to explore recent advancements. This process leads to a critical analysis of innovations and potential future developments. The International Symposium on Achievements, Milestones, and Challenges in Biomedical and Health Informatics, which was held on 29 October 2022, in Athens, Greece, and brought together experts from the field by invitation, aimed to take a comprehensive and critical look at the timeline of the development of health informatics and analyse the achievements and challenges from different perspectives. The Symposium featured thematic discussions that were carefully organised and moderated, drawing on a prior thematic analysis of expert contributions related to the Symposium’s key topics. The experts gave a particular review [3] of the milestones and failures that consequently impacted the very course of development but, at the same time, motivated enthusiasts to think and assess potentially idealistic achievements in the field [4]. Thematic discussions were further used to streamline research queries for the literature review, driving the inquiry toward innovation and directions for the as yet unresolved challenges of the field.

Having some leaders and pioneers in the field among participants at the Symposium provided a unique perspective on BMHI as a vital catalyst in the achievements in medical research and healthcare applications. We hope to present some critical insights that will contribute to the body of scientific knowledge in BMHI and shape the future of our field, which has always been an important tradition of the field. Talks and discussions, combined with periodical reviews on the progress achieved in the discipline, are already recognised as being of key importance for very timely reflections on both perceived changes and advancements, as well as guidelines for new actions [2,4,5]. The assumption from the beginning of the 21st century that “Our society is continuously being influenced by modern information and communication technology (ICT)” [6], was even strengthened during an international workshop in Germany organised 13 years after, with further discussions of the prognoses of how ICT may be influencing healthcare [4,5]. More and more activities were incorporated into proper education and training, highlighting areas for growth in educational recommendations alongside the development of the discipline (e.g., International Medical Informatics Association (IMIA) initially published in 2000 [7] revised in 2010 [8] and 2023 [9], etc.).

Therefore, our paper has five objectives. First, this work aims to provide readers with a systematic presentation and analysis of recent accomplishments and the milestones achieved, along with challenges that have been conquered, all under human-centric development. Second, it aims to systematise our understanding of BMHI trends by identifying future concern and development scrutiny directions. Third, it aims to summarise the findings across the five following key dimensions, which were developed through the thematic analysis conducted: informatics in biomedicine and healthcare, health informatics data, nursing informatics, education and accreditation in health informatics, and ethical, legal, social, and security issues. Fourth, another aim is to critically analyse future directions and innovations, driven by the pandemic’s emerging needs, such as artificial intelligence, machine learning, telemedicine, telemonitoring, wearable devices, and mobile health, as well as the modern clinical approaches, focusing on patient-centric approaches, patient engagement, patient empowerment, and personalised and precision medicine treatments. All of these fall under the umbrella of ethical and security concerns. Fifth, it provides concrete recommendations for improving education and developing experts. By specifying dimensions to present the results, we employed a comprehensive narrative presentation of literature on selected achievements within each dimension.

## 2. Methodology

### 2.1. Overview of Methods for Conducting the Narrative Review

We used a mixed-method approach [10] consisting of a well-established qualitative approach from the literature [11,12] involving the generation of knowledge by involving experts in practical tasks, combined with a quantitative analysis of trends sourced from a review of the literature, which is a widely used technique for systematic literature review [13]. It is organised in the following step-by-step process:

(step I) **Formulation of Research Task**—Experts and pioneers in the BMHI field are assigned a thematic task focused on the research question: “Analysis of Achievements, Milestones, and Challenges in Biomedical and Health Informatics.” This involves preparing original review papers within their areas of expertise to examine the achievements, milestones, and challenges in BMHI. These papers are then subjected to peer review by three independent reviewers, after which they are thoroughly revised to ensure high quality and to minimise bias across the selected topics.

(step II) **Collection and Thematic Analysis**—Seventeen accepted papers were analysed thematically, primarily focusing on identifying and interpreting key themes within their subjects. The thematic analysis was conducted on a dataset comprising information extracted from the papers, including titles, abstracts, keywords, and the titles of sections and subsections. Those data were analysed in MAXQDA2022 software using the thematic analysis approach.

(step III) **Qualitative data collection from the experts’ discussion**—Twenty distinguished researchers in the field—authors of the accepted papers—were selected for their firsthand experience with the early use of informatics in healthcare and their role in shaping its future development. Each participant presented their achievements, milestones reached, and challenges faced, including those newly identified or yet to be resolved for future generations. Based on these presentations, discussions were organised with all participants and structured around the key themes that emerged from the analysed papers. This process facilitated the further clarification, deepening, and exchange of opinions on the topics identified in the research.

(step IV) **Qualitative analysis of trends in the literature**—Insights from the experts’ discussions helped shape precise research questions and identify gaps around key themes. These refined research questions guided the search strategy for a literature review focused on the most significant developments in the field, published from 2022 to the present.

(step V) **Synthesis of Results and Conclusions**—The thematic analysis results and conclusions from the Symposium discussions are integrated with findings from the literature review on future trends, including innovations, transformations, and challenges. Key insights, including potential discussion recommendations and conclusions, are synthesised.

### 2.2. Data Collection for Thematic Experts’ Opinions

Thirty experts in the BMHI field were invited to contribute in the form of original papers for the review, based on the following criteria: contributions in the field and impacts achieved at national, regional, and global levels while maintaining multidisciplinary and multisectoral pursuits, involving academia, industry, and practitioners, coming from regions of different BMHI progress and achievement levels. In addition to their extensive scientific activities, most of them have served or are currently serving as presidents or board members of leading organisations with international impact, such as the International Medical Informatics Association (IMIA), European Federation for Medical Informatics (EFMI), American Medical Informatics Association (AMIA), etc.

Finally, 25 experts submitted 17 papers, all of which were accepted after a peer-review process and subsequently published in a book [3]. An open dataset was created with the following information extracted from the papers: title, abstract, keywords, and section titles with all sub-sections.

### 2.3. Data Analysis of Experts’ Opinions

The thematic analysis was performed following the recommendations and phases described by Thomas and Harden [14], including (1) line-by-line text coding, (2) the development of descriptive topics, and (3) the generation of analytical themes. Three authors (I-O, E-Z, and J-M) carried out the analysis using the MAXQDA2022 software.

**Figure 1:**
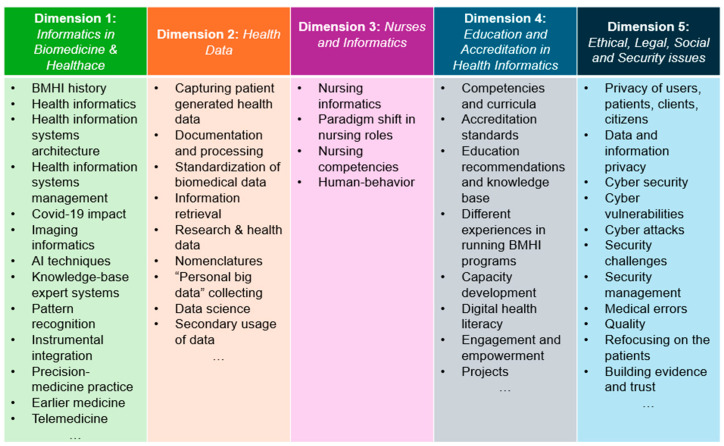
Thematic analysis results: five key dimensions for narrative review presentation.

The dimensions identified from systematic literature reviews [1,15,16] were used for coding and counted only once to avoid data duplication and bias. Additionally, full papers were used for clarification in cases of unclear or missing information. Finally, the raw results of the thematic analysis were further refined through the lens of human-centric development in the BMHI discipline, resulting in five dimensions (Figure 1) for a critical review of the discipline’s development and achievements, as follows:

- *Informatics in Biomedicine and Healthcare*—a dimension that has evolved from the simple application of computers in healthcare to a complete digitally supported transformation and integration of the latest achievements in the field of artificial intelligence applied in the healthcare area;
- *Health Data in Informatics*—a wealth of data that are unrestricted and accessible to a physician, administrators, and patients is revolutionising medicine and still is anticipated to affect the future of healthcare;
- *Nurses in Informatics*—a prominent discipline of crucial importance for the well-being of patients and the development of optimal patient care;
- *Education and Accreditation in Health Informatics*—a dimension crucial for human capital development and raising the awareness and skills of humans, nations, and societies;
- *Ethical, Legal, Social and Security issues*—challenges facing the human perspective of biomedical informatics to ensure full compliance with fundamental human rights and postulates on well-being and privacy.

In this study, a literature review focused on identifying, evaluating, and synthesising relevant literature published in the post-COVID period (2022–2024) that used the most recent developments in the field related to identified dimensions. The search was performed through PubMed, Web of Science, Scopus, IEEE Xplore, and ACM Digital Library databases. The keywords used for the search were a combination of ‘innovations’, ‘patient-centric’, ‘future of healthcare’, and ‘advancements’, with the titles of each defined dimension. The studies highlighted in this article offer a brief glimpse into this rapidly growing field, sparking anticipation for what lies ahead in healthcare practice in the coming years.

## 3. Synthesis of Results and Conclusions

The synthesis of results and conclusions is a crucial step in our qualitative research, involving carefully integrating findings from the published papers and the subsequent discussion organised during the Symposium. In the following subsections, we present this synthesis within each identified dimension, highlighting broader trends and emerging core ideas. We also include divergent views to add depth to the study, showing the range of perspectives and highlighting areas of contention or future research needs.

### 3.1. Informatics in Biomedicine and Healthcare

Experts at the Symposium highlighted that the term health informatics started as medical informatics by Francois Grémy, as in the foundation of the Technical Committee Four (TC4) of IFIP in 1979, which became autonomous and was established a few years before the significant establishment of EFMI (European Federation for Medical Informatics) in 1976. However, the first discussion about medical informatics education took place during a meeting in Lyon in 1974, where the basics of the competencies required by health professionals, mainly doctors, were identified. The evolution of BMHI over the last 70 years is a fantastic story of many successes, a few failures, and some disappointments [17,18]. The discipline of BMHI had to gain its position and acceptance in a very demanding professional area, taking root with clinicians. The health sector is a multi-discipline area; the field, later biomedical and health informatics, gains supporters from all professions working in the health sector, such as information science, computer engineering, scientists in medicine, nursing, biology, computer science, and mainly clinicians.

#### 3.1.1. Health Informatics

All the experts agreed on the importance of highlighting key historical milestones in the development of the BMHI field to foster a proper understanding of its multidisciplinary nature, its ties to healthcare advancements, and the societal challenges it faces. BMHI worked successfully and tackled the field’s requirements regarding its theories, analogies, scientific methodology, semantics, and terminology. The primary target was to populate the hospital information system with data from the medical records. In parallel, the evolution of physics, electronics, transistors and, consequently, computer technology, was changing at a very fast pace. Initially, large mainframes were introduced, followed by personal computers that had low capabilities but were worth using. The introduction of micro-computers in the eighties changed this, leading to personal computers for almost everyone [18].

Furthermore, the European Commission planned and launched an Advanced Informatics in Medicine (AIM) research framework programme to introduce and expand medical informatics in the mid-80s. The AIM programme capitalised on a sudden phenomenon in an era with grander-scale follow-up programmes, establishing BMHI as a crucial and necessary aspect of the European continent. The programme emphasises the role of information and telecommunication technologies in the healthcare sector. The European Community acknowledges the significance of information systems in healthcare. There is a substantial investment towards developing medical and health information systems to support professionals in every health speciality [18].

Several of the invited experts particularly emphasised that health informatics plays a vital role in the field of medical imaging. During the last few decades, various novel digital image processing methods, core or case-specific, have been applied with extensive, good results, formulating the engineering field of biomedical and imaging informatics [19]. The distinguished professors initially delved into health informatics during their doctoral studies in image analysis and pattern recognition. This introduced them to medical informatics from the perspective of imaging and artificial intelligence [18,20,21,22,23]. The applications are vast, from diagnosis, prognosis, and disease management up to precision medicine practice. Medical image processing assists in the interpretation of digital or digitised medical images as evidence and, consequently, generates associated actionable knowledge to achieve precision medicine practice [24,25,26,27] or develop imaging-based, clinical decision support tools [28,29]. As a more specific example, the emerging sub-field of radionics provides a solution for non-invasive tumour characterisation by converting medical images into mineable data by extracting quantitative imaging features. For this reason, the radiomics domain offers an innovative and exciting opportunity in medical imaging informatics, with much potential for achieving precision medicine goals [30].

The potential of digital image informatics in diagnosing, treating, and predicting outcomes significantly improves the ability to enhance patient care and achieve successful therapy and cure. It also facilitates better-informed discussions between healthcare providers, patients, and caregivers in terms of addressing the challenges posed by various diseases. As a result, “imaging informatics” is becoming a potent tool in the array of BMHI technologies, helping us combat diseases more effectively [31].

In conclusion, the significant challenges BMHI faced were its introduction and implementation in the clinical domain and the necessary educational initiatives. In the early stages of BMHI, digital literacy, in the general population and health professionals specifically, was the biggest obstacle for the sector to progress, noted as one of the initial challenges. Furthermore, an initial mistake of the BMHI professionals was viewing the sector as another technological application, and the health professionals believed that BMHI professionals were only technicians that support the new informatics appliances. The reality meets this belief in the middle and proves that it is a multidisciplinary sector. The specialists at BMHI recognise that designing and implementing health information systems is very demanding task. It requires adherence to various parameters and international standards. This includes implementing electronic patient records, addressing security and privacy issues, and navigating ethical and legal considerations related to telemedicine applications. It also involves utilising an intelligent black box approach [6,12], as well as other societal parameters that affect implementations, like a lack of funding, poverty, and cultural obstacles [2,4,32].

#### 3.1.2. Artificial Intelligence

Experts emphasised artificial intelligence (AI), tracing its origins and examining its cross-disciplinary evolution. AI is supposedly the most promising technological achievement, able to solve almost any difficulty and problem in every area of the natural world and probably the most important after the internet in the 21st century. Machine learning (ML) is considered the “heart” of AI, and it is a scorching topic in all research fields dealing with data analysis. ML-based AI is the present and the future. In the health era, most of the issues are highly important and complicated, since clinicians are not able to fully understand the actual workings of the human body in every situation [33]. The term “Artificial Intelligence” is credited to McCarthy, who is said to have coined it during the Dartmouth Summer Research Project on Artificial Intelligence. This project involved the participation of approximately twenty researchers from diverse disciplines, all of whom made significant contributions that have left an indelible mark on history [34]. Alan Turing proposed the Turing Test, which, in addition to its significant role in beating the Nazis, was also the foundation for machine learning as a human and a machine in conversation [35].

A very initial stage of AI in healthcare and collection on the Web is illustrated by the “Greek Oracle”. The vision for decision support in the field of medicine involves the integration of advanced methods and computing techniques to accurately represent the expertise of physicians. This initiative aims to develop tools to effectively enhance healthcare services’ quality [14,36]. These efforts have led to unintended effects, primarily an increasing burden on healthcare providers, leading to a high workload in interacting with EHR at the cost of patient time [37,38,39,40,41,42]. In a 2018 national survey conducted at Stanford Medicine, over 500 primary-care physicians (PCPs) were asked about their use of EHRs. The survey revealed that while the application of decision support in EHRs positively impacted their work, it came with high costs and did not yield the expected outcomes [43]. Based on Karl Popper’s ideas, any complex system can be sliced into small, easily understandable components [44]. As a result, decision support systems have been built according to a set of rules, experts’ knowledge, knowledge databases, observations, and understanding of general medicine metabolism, systems which were impossible to update due to the vast amount of new knowledge and new information, in other words, the big data format of the healthcare era [45,46,47]. AI significantly influences the health sector with many applications based on algorithms, tools, and automated processes from digital image processing [48] to thyroid malignancy treatment under Fine Needle Aspiration (FNA) analysis [49]. AI has broad use throughout health systems, including decision support systems, knowledge, and rule-based support CPOE worldwide.

A central issue for health treatment is the management of the individual differences in pathophysiology, metabolism, genomic, vital signs/activity, etc., and consequently, how all those parameters affect the personalised reaction to the treatment of every disease. The concept of “precision medicine” promises that it will solve the individuality problem in health practices [50]. The considerable number of resources ensures at least a minor positive impact on quality of life. That is the reason why a promising branch of “precision medicine”, called “precision health”, needs the use of adequately designed ML and AI systems [32].

Among the first applications of AI was medical imaging with a state-of-the-art imaging technology process, algorithms, algorithmics, a novel computational analysis based on primitives of machine learning, like decision trees, and novel improvements, like the deep learning of artificial intelligence. From a clinical point of view, this is a powerful driver for achieving precision medicine through quantitative imaging informatics [31]. This quantitative analysis aids diagnosis based on biomarkers, aids surgical decision making and therapy planning, and supports early indications of diseases, helping clinicians respond on time with the proper therapy [24,51]. Even more research has shown ML-based AI applications analysing tweets for Long COVID experience after recovering from COVID-19 [51]. Another example of AI application is research on chatbots, which integrate AI and natural language processing (NLP) technologies to simulate human conversation, as well as its particular use during the COVID-19 pandemic to support healthcare procedures and systems [52].

Interoperability is the cornerstone of decision support [14]. Interoperability pertains to the extent to which a software system, device, application, or other entity can seamlessly connect and communicate with other entities in a synchronised manner without requiring intervention from the end user. This concept is often associated with aspects such as data access, data transmission, and cross-organisational collaboration. In every modern system, interoperability is a must. Electronic health records or other health information systems worldwide are ineffective or unsuccessful because interoperability is still a “dream” for them. Interoperability has the potential to “glue” together different data sources, modalities, and even approaches. This is not easy to incorporate, but is the seed for healthy growth for a correct decision-making process, equivalent to producing decision support systems. The future of decision support in healthcare is built because there is no one-fits-all solution. Each problem must obtain the most appropriate answer based on personalised decision support, supporting the future of customised health.

#### 3.1.3. Progress Achieved, Landmarks, and Future Concerns

The progress achieved in BMHI includes successfully populating hospital information systems with data from medical records, introducing and expanding medical informatics in Europe through research programmes, advancements in medical imaging through digital image processing, and imaging informatics in diagnosis, treatment, and prognosis.

BMHI made significant progress meeting the field’s theoretical, methodological, and terminological requirements. The main aim was to incorporate data from medical records into the hospital information system. At the same time, rapid advancements in physics, electronics, and computer technology, from mainframes to microcomputers, led to the widespread use of personal computers by the 1980s. 

Concerns refer to the challenges and issues faced in the field of BMHI. Experts mentioned the whole spectrum, including the introduction and implementation of BMHI in the clinical domain, the need for educational initiatives, digital literacy among health professionals, and the misconception of viewing BMHI as solely a technological application. Other concerns involve international standards, electronic patient records, security and privacy concerns, ethical and legal matters in telemedicine, and societal parameters that affect implementation. AI is also a significant concern in the field, with its potential to solve various difficulties in healthcare and the need for personalised decision support. Interoperability, or the ability to seamlessly exchange data and information between different systems, is another important concern in BMHI.

A literature review on biomedical technology and health informatics in reshaping healthcare delivery, enhancing patient outcomes, and addressing contemporary challenges in the field highlighted the following key directions:

∘ *Telemedicine*—evolving post-pandemic new trends in remote care encouraging integration with wearable devices [53], the remote managing of various health conditions [53], maintaining continuity of care for chronic diseases [54], acute medical events [55], oncological patients [56], etc.
∘ *AI for patient empowerment*—AI plays a crucial role in assessing an individual’s risk of developing the most common chronic diseases [56], transforming patient-centred care [57]. It particularly empowers patients by improving access to personalised information, care decisions, and equity in healthcare [56,58], alleviating current challenges in optimising clinical care [59]. AI and robotics have been identified as possessing promising potential for enabling independence and enhancing the quality of life for older adults [58].
∘ *IoT, mobile health, deep learning, and blockchain have become crucial technologies in ensuring secure and safe healthcare*—These are positioned to change the landscape of medical diagnosis and treatment [60]. Healthcare 4.0 is a relatively new term that has evolved from Industry 4.0 to meet diverse requirements in the healthcare domain, all aimed at improving patient experience, health promotion, cost control, and clinical satisfaction [61].

### 3.2. The Health Data

Digitisation offers greater access to an expanding volume of data, raising the potential for significant enhancements in decision support [14]. However, collecting massive amounts of data originating from different sources is not enough; the pathway leading to actionable knowledge is led by IT and data science exploitation [31]. The COVID-19 pandemic has underscored the importance of multi-institutional data-sharing strategies capable of addressing numerous challenges [62], proper mining, and generating meaningful actions.

#### 3.2.1. Electronic Health Data Handling

**Figure 2:**
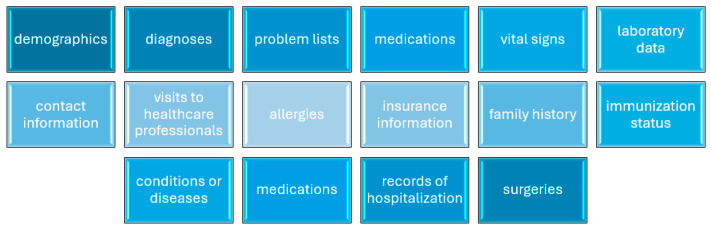
Information that EHR can include.

The electronic health record (EHR), also known as the electronic medical record (EMR), is a comprehensive lifelong repository of health-related data and documents, encompassing various types of patient-specific information, including demographics, diagnoses, problem lists, medications, vital signs, and laboratory data [63,64] (Figure 2). EHRs are inherently complex information systems, posing challenges in design and evaluation [65]. All experts agreed that despite the extensive adoption of electronic health records (EHRs), research indicates that the healthcare system is tapping into only a fraction of their potential benefits [66,67].

While patient care remains the primary focus, EHRs also fulfil numerous other needs, including resource planning, regulatory documentation, quality assessment, certification and accreditation, billing, clinical research, and public health [14]. Indeed, as Moore et al. [68] highlighted, EHRs and health information systems serve a dual purpose. They are designed to offer adaptable and resilient point-of-care solutions while serving as valuable real-world data sources to support clinical research [62].

Experts also suggested several crucial issues that must be tackled to attain such ambitious objectives. Firstly, the current level of interoperability, encompassing both syntactic and semantic aspects among EHRs, remains significantly inadequate. This issue is not primarily due to the absence of suitable solutions [69,70] but rather stems from a combination of factors. It includes an underestimation of the importance of interoperability during the design of EHR systems, at times influenced by vendor lock-in policies [71], and a lack of adequate methods to describe and formally represent healthcare processes and their associated information requirements. It is worth emphasising that the COVID-19 emergency has further compounded this issue, as hospitals and healthcare providers were forced to change their organisational structures and care workflows [62].

The evidence shown in [72,73,74] indicates that healthcare professionals’ perceptions of the usefulness of EHR systems can vary based on their specific usage objectives, context of use, and personal backgrounds, encompassing their competencies and prior experiences with the system. Furthermore, the imperatives to enhance the efficiency of care processes, resource allocation, and billing procedures lead to health providers’ dissatisfaction, reported as a heavy workload driven by extensive EHR interactions, which can come at the expense of the time that could otherwise be devoted to patient care [14,75] (while physicians reported this issue significantly more frequently than nurses [76]); additionally, there is a lack of support for collaboration and intuitiveness of user interfaces [73,74], and nurses have expressed dissatisfaction with the documentation tools [77], highlighting the need for enhancements in areas such as individualisation, self-explanatory features, and error tolerance [78].

Several experts also doubt that EHR systems’ usability appears to differ more based on the specific EHR brand and employment sector rather than a general preference by one professional group over the other. Therefore, when developing EHR systems, it is crucial to consider the viewpoints of these two primary user groups and their unique working environments [65]. In summary, decision support within EHRs has delivered significant benefits, albeit at a substantial cost and without realising its complete potential [14].

#### 3.2.2. Data Science in Healthcare

Recent technology advancements have resulted in powerful “deep learning” tools manageable by individuals from various backgrounds, thus enabling the processing, analysis, and learning from massive datasets containing billions of data points and hundreds or even thousands of dimensions, all without the need for intricate and costly infrastructure [79]. The success of these tools hinges on our capacity to derive meaningful insights from extensive health datasets, ultimately leading to a better comprehension of human health [80].

Experts have agreed that throughout human history, and in medicine, intricate connections have existed between beliefs and scientific understanding. These connections reflect human longing, often rooted in the realm of the divine, and the practical realities of life, which present us with challenges inherent to our human condition [14].

Various complex challenges significantly affect the essential function of informatics in gathering and managing substantial amounts of data, organising, both quantitatively and qualitatively analysing, classifying, and disseminating evidence [31]. The concept of normality plays a pivotal role in medical science, guiding efforts to comprehend the causes of deviations and develop methods for their detection, prevention, and, ultimately, correction [14]. On the other hand, many medical problems are multi-variate under high levels of uncertainty and complexity. The human body’s functioning has not been entirely determined due to individual variations in metabolism, and pathophysiology remains incomplete. The reasons individuals exhibit distinct differences in factors such as drug response and disease risk are not yet fully elucidated, specific to their living context and local information, such as a priori probabilities for Bayesian evaluations. Furthermore, the decisions might differ regarding potential effects, such as their impacts, the resources required, the time, the cost, etc. [14].

Even for complex and comprehensive EHR data, efficiently converting patient data from its raw EHR format into a machine-learning-friendly representation is crucial. This process transforms patient data into meaningful information that can be comprehended and processed algorithmically [81,82]. It further leads to interoperability requirements, crucial for integrating diverse data sources, modalities, and types within a complex temporal framework [14], with standardisation as an essential component in data collection [83].

As challenging as it may be, scientists make significant promising contributions. For example, imaging informatics plays a vital role in interpreting image-based clinical data, thereby enabling the generation of actionable knowledge that can be applied in precision medicine [24,31]. Imaging procedures, frequently non-invasive and associated with minimal side effects, yield comprehensive data encompassing structural, compositional, and functional insights into the human body [76]. This extensive information aids in the characterisation and personalised management of disease for individual patients [31].

Several experts highlighted that the analysis of extensive medical datasets promises to uncover novel and previously unknown connections, patterns, and trends within the data. These discoveries have the potential to catalyse numerous scientific breakthroughs in understanding the causes, categorisation, diagnosis, treatment, and progression of diseases. Such breakthroughs may involve the development of computational models that utilise data to accurately predict clinical outcomes and disease progression, ultimately enabling the identification of individuals at high risk and the prioritisation of early intervention strategies [84]. Additionally, these analyses can help evaluate the impact of public health policies using real-world data [85]. Nonetheless, the realisation of these ambitious objectives still faces numerous challenges [80].

#### 3.2.3. Progress Achieved, Landmarks, and Future Concerns

The progress achieved in health data in informatics can be easily presented as a pathway from the digitisation of processes in healthcare to the generation of electronic health records as a comprehensive lifelong archive of health-related data and documents containing diverse patient-specific information, which significantly exceeds the medical data on diseases and treatments. However, this resulted in a massive volume of data, which opened up great potential for various improvements in healthcare systems’ functioning processes and procedures.

The key milestones refer to data collection and generation of EHRs and the further transformation of EHR data into formats understandable for machines and their reasoning capabilities. Therefore, the momentum in EHR development includes a period of dual and parallel use of data, one for improving current procedures and processes and the other for better understanding human health and generating new hidden knowledge.

The concerns refer to the challenges and issues faced with the extensive adoption and utilisation of electronic health records (EHRs), as well as the particular challenges of data-sharing and cooperation during pandemics such as COVID-19. Despite the large use of EHRs, not all of their potential is used, as a result of challenges in technological solutions and the lack of acceptance by medical and professional staff. However, researchers and innovators are making significant progress in further developments, while human-based challenges mostly remain the same while simultaneously motivating for further advancements.

A literature review on health data as a transformative source in healthcare, driving innovations shaping future developments, highlighted the following key directions:

∘ *Wearable technology for continuous health monitoring, personalised healthcare, and real-time data collection*—enabling the real-time monitoring of health parameters [86], improving early illness detection and management [87], with the potential to enhance occupational health and safety [88].
∘ *Big data mining electronic health records combined with other data sources*: this includes social media mining for health insights [89], natural language processing for mining case reports and other texts for social determinants of health [90], tracking disease outbreaks and understanding population health dynamics [91,92,93], patient-reported outcomes collected through surveys and mobile applications providing valuable insights into patients’ health status, treatment satisfaction, and quality of life [94].
∘ *Precision medicine*—targeting treatments to individual genetic and clinical profiles benefiting from AI, promising solutions for creating realistic, privacy-preserving patient data [95], with the internet of medical things (IoMT) supporting real-time digital health services for in-hospital and out-hospital patients [96], as well as precision-centred treatments of specific diseases, including cancer [97], mental health [98], noncommunicable chronic disease [99], etc.

### 3.3. Nurses and Informatics

Numerous experts have invested substantial effort in analysing the essential role of clinicians in the healthcare system, as well as the critical involvement of nurses, who constitute the largest group within the healthcare workforce. Clinicians and nurses are the main actors in the sustainability of healthcare systems. Their role affects all levels of care, from primary to specialised care, all age groups, from newborn to elderly care, and all domains, such as preventive, reparative, rehabilitation, and palliative care [100]. In addition, the incidence of non-communicable and general infectious diseases is increased in ageing populations, mainly in developed countries. Health coverage is a universal need and a primary global concern for healthcare systems, especially if we add in the factor of globalisation [101]. Clinicians are close to patients and primary care. In addition to that, nurses are also close to the population and the patients in a different way, and they are the ones with whom patients have closer connections. Nurses’ role is to improve health outcomes and reduce disparities [102], communicating with patients and acting as the intermediary among the clinicians and other healthcare professionals. Nurses also miss breaks and leave late, sometimes pulling 14 or 16 h shifts, forced into overtime. The focus is on flow, stabilising the patient, and moving them through the system as quickly as possible. Clinicians and nurses are not heroes; they are human, and they are exhausted.

#### 3.3.1. Nursing Informatics

Technology affects the stamina of nurses, and the extended use of technology in the healthcare era emerges as part of the essential role of nurses for the future of biomedical and health informatics. Holistic and applied critical thinking is a must as a tool to tackle rapid technological development, which, on the contrary, can improve healthcare delivery based on innovative approaches, and nursing has a unique role within that. For the full utilisation of “smart” systems in healthcare delivery, the cultivation of digital literacy by nursing staff is currently needed, in the form of adaptive and scientific skills developed for technological advances [100].

Due to the bad working conditions, there is a severe nursing shortage, which is not new. The most critical need is often hospital-based nursing, where nurses are not at the bedside, or there are too many patients per nurse. According to research in Arizona, 31.36% of hospitals are experiencing critical staffing shortages; according to Becker’s Hospital Review, the COVID-19 pandemic only exacerbated the drought, causing more nurses to burnout and eventually leave the industry. The WHO reported the chronic shortage [103,104].

The influence of technology on human behaviour encompasses the interaction of human factors, computer interaction, and the other effects of technology on well-being, including the impact of robots and AI-based systems on nursing care. It is crucial to develop nursing competencies at all educational levels to nurture a mindset and culture within the healthcare workforce that is equipped for a digital health system. Educational tools such as gamification and simulation can help prepare educators to train healthcare professionals and researchers who play a pivotal role in bridging the gap between technology and practical application [100].

The foundation and evolutions of nursing informatics (NI) came from a nurse’s need to tackle the challenges of nurse discipline and deal with the opportunities of clinical information storage, handling, and retrieving, utilising health information systems to address real work problems. It is worth mentioning that nurse informatics amplified its progress due to nurse professional organisations in the early 1970s [105]. Due to nurses’ involvement, essential aspects such as the standards for clinical language and data limited the functionality and usefulness of the early applications included in the clinical era discussion for health informatics. As a result, the first steps for designing every modern information system became noticeable and understandable, because nurses were very aware of the lack of essential “seeds” [105].

The nurse’s key role in the healthcare system is to be actively involved, a role that is essential for proper and appropriate technological health development. The end users of the designs are mainly nurses or other healthcare professionals closely collaborating with a nurse during the use of an information technology system, and thus, understanding the end-user perspective; nurses are the core population, which is a critical factor for the success of the system [106].

One of the most conflicting information technology applications is tele-nursing, a part of the general term telehealth. The technology of telehealth is not new, but since hospitalisations and remote treatment emerged during the COVID-19 pandemic, there are various cases in which the technology has been used out of necessity. Telehealth utilises remote conference and remote signal (vital signs and images) monitoring and transferring. Solutions such as phone calls or video conferences are those used to help with patient care. Through telenursing, telemonitoring, or remote patient care, nurses can monitor patients from outside the patients’ hospital rooms or at the patients’ homes and still respond effectively to patients’ needs. Cases like the Memorial Hermann Health System in Texas used virtual nursing during the last pandemic. When patients feel sick, telenursing can save them a trip to the emergency room (ER) or hospital. The Nurse On-Call programme at Banner Health helps to keep people out of the hospital, only focusing on the patients and making care more convenient for them [107].

Following the experience of a pioneer in the BMHI specialised in nursing informatics, we must acknowledge the foundation and expansion history of the Nursing Department of the University of Athens, related to the discipline of the health informatics laboratory, led by Prof. Mantas [18]. The laboratory has experience in introducing the early stages of informatics to nurses—with a ZX Spectrum microcomputer connected to a TV set—to teach the nurse students the fundamentals of computer composition and functionality. This is followed by using a BBC micro as the next microcomputer. In parallel, all machine-power-demanding research was performed based on mainframe computers such as the CDC Cyber 6600, 7600, and later 172. The most demanding part of this achievement, as Professor Emeritus Mantas described in [18], was to convince the IT experts of the University that the Nursing Department needs such access; this underscores the problem mentioned above in terms of the IT sector understanding the multidisciplinary nature of the BMHI [16]. Furthermore, the Health Informatics Laboratory in the University of Athens Nursing Department, led by Professor Emeritus Mantas, has been involved in various EU research projects and supporting the European Commission’s general aims, planned and launched initially with AIM and other health-funded programmes. It is worth mentioning the iconic non-educational projects like EIPEN, which supports interprofessional activities within the healthcare institutions, and the EUnetPAS project, which aims at improving patient safety conditions in healthcare institutions across Europe by minimising medical errors [18].

#### 3.3.2. Nurses in the Future of Biomedical and Health Informatics

The globe is facing a changing environment, due to climate change producing massive fires and floods, regional wars, expanding migration, and pandemics; additionally, nursing is facing a worldwide workforce shortage, affecting the quality of health provision. Information technology offers promising support for nurses who encounter some difficulties. Applying information technologies into clinical routine must be considered an essential step to support improvement, not as a replacement for professionals. Potential applications for robots, like the ones used in robotic surgery, are serious games [108,109].

The aim of such modern information-technology-based clinical applications should be to promote health provision in terms of practice and quality (security, efficiency, equity) and, secondly, to save money [110,111]. According to the WHO Global Strategy, there are various goals for nursing and midwifery in 2021–2025 to reduce nurse burnout. The use of technology is rarely proposed as a means of improvement [100].

According to the study of Professor Patrick Weber [100], information technologies could provide solutions to nurses in various cases, such as:

- Interdisciplinary/inter-professional/patient partnership;
- Interoperability;
- Time-consuming activities that are not clinically focused;
- Undertaking administrative, coding, and organisational activities, and rules;
- Simultaneity events constantly occurring at the clinical and organisational levels;
- Support patient management, logistics, and the communication of any information and data needed, and support the continuity of healthcare provision to the patient, all along the clinical pathway and between shifts;
- The safety of the patient;
- Predictive and prospective parameters [100].

The role of nurses is vital throughout the healthcare system. The new challenges of passive monitoring and wearable technologies seem to be the present and the future of patient monitoring, which in other words is the main work of a nurse. The continuous measuring, behaviour, and physiology is the work that every nurse does within a hospital or in any other nursing intervention, such as in a school, in a penitentiary, in primary care, prehospital, etc. Technology can continuously measure almost all aspects with wearables and other sensors, which all provide the ability to capture the behaviour and activities of daily living.

Personal health informatics (PHIs), is defined within consumer health informatics as “any electronic tool, technology, or electronic application that is designed to interact directly with consumers, with or without the presence of a health care professional that provides or uses individualized (personal) information and provides the consumer with individualized assistance, to help the patient better manage their health or health care” [112].

This can be translated to removing the need for nurses and other clinicians’ to be present, without fully replacing them. The remote monitoring tools gather data about the health status of the person under surveillance and they can share these data to other related healthcare professionals [113]. Leveraging remote monitoring systems empowers patients to actively participate in their own healthcare delivery and disease prevention. These systems encompass a wide range of platforms that can facilitate the capture of personal generated health data, including paper-based tools like diaries or forms, as well as wearable or implantable devices. The tools are of various styles and types, consisting of capturing and storing data, supporting clinical decisions, providing alerts, predictive analytics tools, machine learning, pattern identification, and physical language analysis. The list is numerous and incorporates each system, facilitating an entire arsenal for a continuous monitoring and portable personal health record.

The examples for personal health informatics applications are numerous. Referring to some of them as categories of the BHMI, a subsection of PHIs known as the digital phenotyping is one category, which involves the use of smart phones for tracking movement, time spent online, and even engagement in social interactions, as a “moment-by-moment quantification of the individual-level human phenotype in situ using personal digital devices” [114]. Phenotyping can exist in the form of passive implementation without user involvement, like the use of a GPS sensor.

Digital phenotyping systems can be further categorised into content-free patterns (for example, capturing reaction time for tapping, scrolling, and typing) or “content-rich” systems (such as analysing social media postings, voice recordings, or one’s search history). Digital phenotyping systems sometimes integrate wearable biosensors [115]. The use of wearable biosensors further expands the applications, including such systems as monitoring real-time drug use [91] and alcohol consumption [92], capturing and analysing autonomic nervous system activity through electrodermal activity, 3-axis acceleration, ECG, and temperature, to support cognitive behavioural therapy (CBT) [116].

Personal health informatics applications in smart homes are widely applied and vital for many health issues. The use of sensor technologies in a home is not new. In the late 1990s, sensor technologies were explored to enable passive monitoring in homes, primarily for older adults and individuals with disabilities. Using sensors allows for the transformation of a home into a “smart home”, a safe environment for health and well-being. The explosion of the internet in the early years of this century facilitated a new perspective on smart homes, with applications based on Internet of Things (IoT), interconnected devices that can be controlled remotely [113]. The EU community funded various research projects in the field of smart homes, such as the ENABLE project, supporting individuals dealing with early-stage dementia [117], and the PROSAFE project, monitoring residents’ movements and detecting potential falls [118]. In addition to this, other research teams such as Chung et al. [119] conducted a study on smart home technology use among Korean American older adults, while Gaugler et al. [120] examined the intervention called Sense4Safety [98], which utilises smart home tools to provide nursing intervention for socially vulnerable older adults [121].

#### 3.3.3. Progress Achieved, Landmarks, and Future Concerns

The progress achieved in the healthcare workforce’s adoption of health informatics covers various topics worth mentioning. Various information technologies supporting nurses and clinicians are widely established in healthcare daily practice, supporting patient management, the continuation of healthcare provision to the patient, and clinical pathways, as well as accountability between shifts. The health informatics applications support the main aim of the healthcare workforce, which is patient safety. Furthermore, clinicians and nurses are supported by informatics to improve the required modern capabilities regarding the interdisciplinary aspects, inter-professional cooperation and patient partnership. The close involvement of nurses is a great achievement in technologically advanced countries, which is a critical factor for the success of the application of information systems. The COVID-19 pandemic incentivised the healthcare workforce to incorporate telehealth and tele-nursing applications on a worldwide scale. In addition to the changes in educational methods and tools, due to the pandemic, the healthcare workforce was driven to the further utilisation of gamification and simulation learning.

The main landmark achievements regarding the use of health informatics by the healthcare workforce are numerous. In historical order, the first worth mentioning is that health informatics was pushed by the nurse professional organisations in the early 1970s. More than worth mentioning is the fact that among the pioneers of Nursing Informatics is Professor Emeritus John Mantas on the Nursing Department of the University of Athens. Furthermore, essential parts like standards and clinical informatics language are established due to nurses’ involvement and efforts. The extension of telehealth, telemonitoring, telenursing and mobile health applications during the COVID-19 pandemic is among the major milestones for health informatics.

Experts agreed that the clinicians’ and nurses’ use of health informatics faces many future challenges. Although the technology is closely related to future changes, the main challenge regarding the full utilisation of health informatics is the cultivation of digital literacy by healthcare professionals, nurses included. In addition, information technologies need to be incorporated into clinical routine, and the wider application of robots and 3D printing is a needed added value for the healthcare sector. Furthermore, interoperability solutions need to be extended to the worldwide health information system to confront the upcoming pandemics. Finally, the emerging technologies in personalised health rewrite the rules of patient safety.

A literature review on the role of technology in enhancing patient care and nursing practices is highlighted the following key directions:

∘ *AI-assisted care coordination and workload management*—AI-enabled robotics and telehealth solutions expand the reach of nursing care [122], improving the accessibility of healthcare services and the remote monitoring capabilities of patients’ health conditions [123], thereby fostering a more efficient, data-driven, and patient-centred approach to care as a vital area covering a wide range of topics for innovations, including patient satisfaction rate, patient involvement, nurse–patient communication and collaboration [124], pain assessment, management [125], etc.
∘ *AI-powered clinical decision support systems*—improved nursing efficiency, accuracy, and clinical decision support by leveraging AI-driven technologies, such as natural language processing and machine learning [126].
∘ *Preparing nurses for the future*—AI’s practical applications, ethical considerations, and its role in enhancing nurse–patient interactions [127] and nursing education programmes for a future-ready nursing workforce capable of utilising cutting-edge technologies to improve patient care and healthcare outcomes [128].
∘ *Collaborative intervention in the era of health 4.0*—collaboration across academic institutions, clinical practice, research sectors, and the business industry is essential to efficiently develop specialised solutions that address the specific needs of individuals [128].

### 3.4. Education and Accreditation in Biomedical and Health Informatics

BMHI is a multidisciplinary field that demands that practitioners and researchers possess expertise in various domains, including in the fascinating realms of biomedicine, healthcare, computer science, mathematics, data science, and machine learning, while widely acknowledging the necessity of effectively managing interpersonal relationships and organisational challenge [129,130]. Nevertheless, experts noted that BMHI is more than the mere amalgamation of various disciplines; it encompasses distinctive facets that emerge at the crossroads of these fields [130].

The importance of education and capacity development to reach the BMHI demands can be illustrated with “What we are, what defines us, is mostly the accumulation of the efforts of others who influence us”, said Mantas in [107], even reflecting nursing education, but this is general enough for highlighting the compassionate approaches dedicated to responsible digital transformations and innovations in health and contemporary healthcare [131].

#### 3.4.1. Biomedical and Health Informatics Education as a Change Agent

The core of any capacity-building endeavour revolves around individuals. Facilitating opportunities for people to acquire knowledge and understanding is crucial for evaluating and fostering the adoption of any concept. The growth of health and biomedical informatics education has typically mirrored the advancement of information management systems and the integration of digital tools and services in healthcare [131,132]. The COVID-19 pandemic showed how dynamic progress can be accelerated in the field of BMHI, which directly leads to the required alignment of educational activities with present requirements and forthcoming advancements [9]. Societies become more complex, and we entirely rely on the infrastructure that is assumed to work ideally, which does not correspond to reality.

Therefore, the BMHI education is expected to contribute to the creation of new forces that will be capable of responding to new challenges and leading developments and innovations in the field, as well as to provide training to both healthcare and informatics professionals to work together in developing, implementing, and evaluating healthcare and public health.

Starting in the early 1980s, the whole spectrum of different study programmes has been developed and implemented at national/regional levels [133,134,135], designed to serve citizens and professionals according to the BMHI needs and requirements of the nations. From another perspective, the rapid technology development and its applications in various fields have made it imperative for BMHI education to align with the latest global trends and innovations in the field of technology-enhanced healthcare [9].

However, ongoing evidence and reported experiences [136,137,138] underscore the robust connection with the support of policy and decision-makers at the national level as an essential driver of a two-way coupling: societies can directly support broadening the field and creating opportunities to develop educational components, while the natural development of education implicitly supports the development of culture in the area [8].

Further, education development, regardless of the field, illustrates the power of knowledge and advancements within each society/nation. This is particularly highlighted when dealing with the effective coordination and delivery of healthcare and information exploration, utilisation, and improvements, which BMHI brings about.

Keeping this in mind, the development of the BMHI profession as a process of “planting and cultivating the seeds” [131] shows different experiences in different countries, with some still struggling with the characterisation of the BMHI workforce and experiencing significant changes in the healthcare environment (e.g., the shifts in population health needs coincide with the growing importance of universal health coverage, etc.). However, significant shifts in the demands for delivering effective and efficient healthcare have been documented at the global level, also creating a substantial disparity between the workforce’s current capabilities and the systems’ overall readiness.

In response to modern challenges and demands, experts emphasised that BMHI education fosters literacy development, proficiency enhancement, and the expansion of skills among professional constituencies. As a catalyst for change, it benefits citizens, the ultimate beneficiaries of healthcare services and all healthcare professionals and societies within modern healthcare. The creation of young generations in the field of BMHI is still one of the desirable and essential needs in many regions at the European and global level, which can be tackled by academics and professionals by prioritising the transfer of experience and best practices from those at the higher levels of BMHI education and societies in general.

#### 3.4.2. Educational Recommendations, Competencies, Curricula and Capacity Development

Several experts revealed key historical insights into the evolution of BMHI education pathways. In 1983, the first health informatics programme aimed to familiarise people with the diversity of data [18], while the growing advancements in BMHI consequently imposed a variety of disciplines within BMHI and broadened the competencies of educational programmes. The evidence continues to highlight the lack of a fitting approach for the creation of BMHI education [139,140,141], with one perspective focusing on integrating BMHI courses into existing traditional programmes (like medicine, computer science, etc.), and another opposite perspective concentrating on the creation of new BMHI study programmes, and those are implemented as mixed approaches (the creation of modules in final years, etc.).

BMHI education has been designed to cater to various recognised qualifications at different stages of academic career progression, including bachelor’s, master’s, and doctoral levels, with the creation of continuing professional development opportunities primarily intended for practitioners and recognised professionals within the field.

The process of developing education and research programmes in BMHI can be made more systematic by using a two-layered, stratified paradigm. One layer of the paradigm can focus on its historical evolution, moving from country-based methods to computational uniqueness, embracing digital technology and social networking, or transitioning from discipline-specific standards and systems towards global and universal concepts and structures [132]. The second layer of understanding BMHI involves examining the educational needs of individuals in this field. This perspective has influenced how we define the essential competencies for the profession and determines the appropriate curricula to develop these competencies. In BMHI, we have different types of learners, including practitioners, professionals, and what are commonly known as ‘users’ of BMHI, such as clinicians, researchers, administrators, policymakers, and consumers (including patients and citizens). As a result, there is a diverse range of learners, each with unique educational needs and requirements [130].

Several experts elaborated on their own experiences with national contexts and high-lighted how tradition shows that this paradigm is often reinforced by project support, particularly projects such as Erasmus (a programme funded by the European Commission). In 1987, the first Erasmus project in the region provided two phases for funding for MSc programmes in health informatics, which lasted for eight years [142]. The first step involved developing a health informatics curriculum for the MSc level after an international workshop in Athens. The next step involved implementing the programme, which included an exchange between professors and students. Initially, this exchange involved six European universities, but it expanded to 20 European universities as the programme progressed.

Much later, in 2016, the Erasmus programme was used for transferring best practices and gaining experiences from leading European universities to Montenegrin universities and establishing a new MSc programme in health information management [143]. The project was funded for four years, among which one year was used for the implementation of the programme. Additional research projects serve as significant milestones within the research process in BMHI, as well as the most recent attractive dimension of fostering innovations and entrepreneurship in the field [18]. From its inception, the evolution of the educational process can be viewed as an ongoing chain of responses to advancements in the field, driving forces for enhancement, and disseminating practices and knowledge among a network of participants, both at the national and international levels.

#### 3.4.3. Quality Control of Education—Accreditation Process

The accreditation of a study programme is a formal process through which an educational institution or programme undergoes evaluation by an accrediting body or organisation. This evaluation aims to assess whether the study programme meets established standards and criteria for quality and effectiveness in education. Several initiatives to verify formal accreditation have been initiated and are currently in progress [8,9,144].

As of 2022, a distinct need persists for users, generalists, and specialists in BMHI, particularly concerning developing practical guidelines and procedures. These resources are essential to assist educators in the establishment and launch of new programmes, as well as the revitalisation of existing ones within the field of BMHI [132].

This is entirely in line with recent highlights provided by the World Health Organization (WHO), emphasising the widening gap between the current and necessary healthcare workforce to meet the Sustainable Development Goals related to health by the year 2030 [9,145], which is further supported within an introduced strategy aimed at assisting countries in the creation of accreditation bodies [146]. These bodies are intended to ensure high-quality education for healthcare professionals.

Accreditation bodies are generally established at the national or regional level, and are primarily tasked with evaluating and ensuring the quality of educational institutions and programmes. While these bodies may not always prescribe specific academic content or standards for every academic discipline, as experts and institutions typically develop those, they establish general criteria and standards for assessing educational programmes’ overall quality and effectiveness, including BMHI. Furthermore, in the United States, the Commission on Accreditation for HI and Information Management Education (CAHIM) [146] oversees the processes of quality monitoring and programme accreditation as a follower of the American Health Information Management Association (AHIMA), which was created with the mandate of monitoring the programme at various levels [147].

On the other hand, while a programme accredited by a national accreditation committee meets local standards, it may not necessarily adhere to European-level quality standards in BMHI [9]. Therefore, experts highlighted that international accreditation offers a competitive edge to the programme and provides learners and collaborators with valuable insights into its quality. International accreditation can also guide and elevate educational achievements in various nations and reinforce the programme’s position as a recognised national brand [132], supporting global collaboration and sharing courseware initiatives.

The International Medical Informatics Association (IMIA) has published three versions of its international recommendations in BMHI education, initially in 2000, then revised once in 2010 and again in 2023. IMIA accreditation is carried out by the IMIA accreditation body (usually consisting of three experts) and primarily refers to the assessment of the quality of the programme and the fulfilment of the criteria defined by the IMIA accreditation framework. Accreditation is valid for five years, after which the programme can be re-accredited.

Most recently, the European Federation for Medical Informatics (EFMI) launched a committee, AC2, responsible for promoting and fostering the accreditation process of BMHI programmes in Europe. The AC2 accreditation follows the model established by the IMIA in 2010 [148,149]. It strengthens the accreditation process from a European perspective.

The advancements and ongoing enhancements of educational programme should be responsive to societal shifts driven by globalisation, digitisation, and digital transformation, particularly within the healthcare sector. These efforts should prioritise the academic requirements of healthcare professionals, computer scientists, and decision makers seeking to acquire knowledge and skills in BMHI across various levels.

#### 3.4.4. Progress Achieved, Landmarks, and Future of Concerns

The progress achieved in BMHI education is fully parallel with the progress of the implementation of digital tools and services alongside advancements in the information management systems within the healthcare sector. Therefore, there is no unique best-fitting approach for all nations and systems, and it usually includes both formal and non-formal education. However, although it once caused debates throughout history, BMHI, with a multi-disciplinary nature, has imposed multidisciplinary aspects of education, not only for doctors and medical staff but also for other professions, from computer scientists, lawyers, and economists. It is noteworthy that the level of awareness needed for cultivating the necessary skills and enhancing health digital literacy for all citizens has been reached.

Experts proposed key milestones in BMHI education, viewed through the prism of BMHI as a discipline. However, the initial key points refer to the recognition of the need for education and the very definition of the discipline in educational programmes. The next key point includes a multi-disciplinary assessment of educational needs in the profession, while the definition of standards for the development of knowledge and competences is key—not those specific to a nation or system, but at the European and international level. Currently, accreditation standards are viewed as responses to globalisation and the swift digital evolution of the healthcare sector. Consequently, these standards are embraced across various disciplines, undergoing periodic analysis and refinement.

The concerns that BMHI education faces mainly refer to the traditional education systems that usually do not respond quickly to changes and as such reject them by default. Ensuring the quality of the programme, which includes all aspects of the educational process, is an unresolved issue, even if the accreditation standards are settled. In the end, COVID-19 left behind challenges in education as well, and in the implementation process itself, because it imposed the instant introduction of digital implementation, which caused difficulties in implementation of BMHI programmes, and these need to be addressed urgently. The challenges are deeply connected to innovations and evidence-based knowledge, as well as the general coverage not only of workers and professionals, but from children to the elderly, considering that everyone is becoming more and more exposed to digital technologies including digital health.

A literature review on biomedical and health informatics education as a rapidly evolving system to address the future needs of healthcare highlighted the following key directions:

∘ *AI integration in education*—the development of personalised, adaptive learning environments by simulating clinical scenarios [150], AI-driven educational tools providing tailored learning pathways that improve student engagement and academic success [151], and training future health professionals to use AI to enhance patient outcomes [152].
∘ *Reflection of advancements in biomedical and health informatics education and training*—ensuring that practitioners remain at the forefront of medical innovations [153], the integration of cutting-edge biomedical and health informatics methods, emphasising both theoretical knowledge and practical applications [154], and digital health literacy for overcoming the problems and barriers related to the use of digital health applications [155].

### 3.5. Ethical, Legal, Social and Security Issues

#### 3.5.1. Legal and Social Dimensions of Health Informatics

The legal and social dimensions of healthcare informatics pose well-known issues, evident since the first time it was applied. Data protection, data privacy and security have been extensively discussed, and many laws, applications and methods exist in the healthcare area. New applications like personal health informatics and the wide application of AI in the form of clinical decision support systems form part of a new and very demanding situation for healthcare. In addition, studies are required to address the quality, accuracy, and reliability of the data and decisions made in different settings and case scenarios.

The accuracy and reliability of data-capture platforms will need to be examined as more individuals choose to generate and share data with their clinicians. Experts have expressed doubts about many aspects of personal health informatics that remain unanswered, listed as follows:

- Does the use of personal health informatics tools lead to more patient engagement and ultimately patient empowerment [113]? Is this a problem for the proper treatment of patients?
- Is it beneficial for the patient to know the measurements and numbers but not be able to interpret them as a specialist?
- Does this situation affect the performance of any treatment positively or negatively?
- Are there any other consequences of the daily monitoring of personal health using telematics? How are all the personal data protected?

Personalised digital tools must be aligned with the global, regional, or national health system. Each country and each region has its own specificities, and monitoring systems need to follow the laws and needs of various places. The applications are dependant upon the place in which they are utilised. Furthermore, the economic status of potential users is closely related to personal economic status. Moreover, the devices need to have access to telecommunication channels, which also costs money and makes the use of telemonitoring a very expensive application for poorer countries. The COVID-19 pandemic, alongside recognised social crises such as climate change and structural racism, has underscored how technological innovation can worsen inequality by overlooking or perpetuating marginalisation and injustice [113]. According to the American Community Survey (ACS), in 2018, 18.1 million Americans, accounting for 15% of all households, did not have any “broadband” internet service subscriptions [156]. The adoption of those solutions in rural areas faces other major difficulties, although it is much more needed due to the absence of a healthcare workforce.

Furthermore, the accessibility of PHI, and its utilisation by patients or healthcare specialists with various disabilities, such as those experiencing functional or cognitive limitations, visual or hearing impairments, old age, a lack of digital literacy or human network to help them, constitute a great obstacle for a major target group to use those systems. There are technical and legal solutions to support these groups, like closed captioning, screen readers, web accessibility standards, mobile accessibility standards, visual or audio support, and peripheral monitoring devices with accessible design [113].

#### 3.5.2. Cybersecurity of Digitised Healthcare Systems

The healthcare industry has been profoundly impacted by digital technologies, with the advent of modern information systems and the integration of smart devices enabling improved patient communication and greater patient access to treatments [157]. Several key elements of contemporary information systems have had a substantial influence on enhancing the quality and accessibility of healthcare services, including the following:

(i) The adoption of electronic health records (EHRs) instead of traditional paper records, resulting in increased operational efficiency within the healthcare sector.
(ii) The establishment and utilisation of telecommunication networks and services, fostering seamless communication and collaboration between patients and healthcare professionals.
(iii) The integration of mobile health (mHealth), telehealth, and telemedicine solutions, which have not only streamlined patient management processes but have also elevated the overall quality of healthcare services.

However, the integration of advanced technologies in healthcare has facilitated patient data collection while concurrently enhancing the quality of services. Nevertheless, any operations involving personal data entail potential vulnerabilities within the system, stemming from software vulnerabilities, human errors, or security flaws [158].

As a result, the increased number and diverse range of cybersecurity threats have brought about a marked escalation in vulnerabilities and cyber risks for healthcare organisations. Breaches involving sensitive health data can originate from internal and external threats, with the most prevalent including hacker intrusions, unauthorised data access and disclosure, data theft, and data loss, among others. The trend demonstrates a swift upsurge in the frequency of such attacks, with a staggering 162% increase observed over the past three years [159,160].

All experts highlighted that cybersecurity is a mandatory and essential responsibility for all healthcare sector stakeholders, including healthcare service providers, insurance organisations, pharmaceutical and biotechnology firms, and companies involved in medical device manufacturing, software development, and the production of hardware components [161].

Within this framework, the fundamental objectives of safeguarding healthcare organisations in the realm of cybersecurity can be outlined as follows:

(i) Ensuring the availability of healthcare services;
(ii) Ensuring the seamless functionality of medical systems and devices;
(iii) Preserving the confidentiality and integrity of patient and service-related data;
(iv) Ensuring a prompt response and prevention of both external and internal cyberattacks [162].

To effectively combat cyberattacks, it is essential to comprehend the motivations driving attacks on healthcare institutions. The primary reasons behind launching cyberattacks on healthcare systems or organisations include the following [159]:

(i) The immense importance of personal health data on the black market: Patient health records contain a wealth of information, making them a valuable commodity for various forms of data abuse, including identity theft and illicit activities involving insurance companies.
(ii) The strong willingness of victims, particularly healthcare institutions, to pay substantial sums to attackers to release inaccessible patient data and ensure the uninterrupted operation of healthcare services.

Certain aspects of the modern healthcare system’s organisation exhibit corresponding vulnerabilities that cyberattackers frequently exploit to undermine security and functionality. Evidence highlights the existence of different factors [160], such as the vulnerability of medical devices to cyberattacks (due to the lack of a permanent focus on meeting cyber security requirements in addition to specific medical purposes) and their connection to the health organisation network; the necessity of remote access to confidential data (mainly required for medical staff and their readiness for remote monitoring of patient health and teamwork); the reluctance of staff to embrace modern technologies and the low level of their cybersecurity knowledge; as well as health information sharing as an ongoing responsibility.

Cyberattacks are continuously evolving and growing in sophistication with each passing day. Healthcare institutions are responsible for prioritising investments in cybersecurity, which unquestionably protects their patients and their ongoing commitment to providing healthcare services. The wealth of data regarding documented cyberattacks on healthcare organisations should serve as the foundation for distilling vital insights in establishing robust cybersecurity protocols within healthcare institutions [163]. The critical areas that require significant investment and capacity building within a healthcare organisation are delineated as follows [163]:

(i) Equipping employees to confront cyberattacks: employee awareness and cyber hygiene;
(ii) Preparing the organisation to address cyberattacks: policies and procedures;
(iii) Comprehending vulnerabilities: risk assessments and continuous monitoring;
(iv) Establishing a response strategy: training and preparedness, communication and information sharing;
(v) Strengthening cyber infrastructures: access controls, redundancy, patching, and encryption.

Finally, experts highlighted that the information available regarding cyberattacks on healthcare organisations should serve as a foundation for extracting essential insights to enhance cybersecurity measures within healthcare organisations. The process must be continually monitored, evaluated, and adopted to new standards and advancements in the field.

#### 3.5.3. Progress Achieved, Landmarks, and Future Concerns

The progress achieved in healthcare informatics includes advancements in data protection and security, the development of new applications like personal health informatics and AI-based clinical decision support systems, and studies to ensure the quality and reliability of data and decisions in various scenarios. Additionally, the protection of personal data and the alignment of digital tools with different health systems and economic statuses pose challenges. Overall, progress has been made in healthcare informatics, but there are still significant hurdles to overcome.

The landmarks in healthcare informatics include data protection, data privacy and security, personal health informatics, AI in the form of clinical decision support systems, the quality and accuracy of data and decisions, patient engagement and empowerment, data interpretation by specialists, the impact on treatment performance, the consequences of personal health monitoring, the protection of personal data, alignment with health systems, accessibility and usability for individuals with disabilities, economic considerations, and disparities in access to telecommunication channels and broadband internet.

However, experts expressed doubts about several unanswered questions concerning the impact of personal health informatics tools contributing to patient engagement and empowerment, the interpretation of measures and numbers by non-specialists, and the consequences of daily monitoring of personal health using telematics. Technological innovation in healthcare informatics must also address issues of inequality and accessibility, such as the lack of broadband internet service in certain areas and the limitations faced by individuals with disabilities. No less important is the issue of digital health literacy, which is still at a low level of development; on the other hand, the general exposure of all generations to digital services, including those related to the health sector, is increasing.

A literature review on ethical, legal, social, and security issues associated with biomedical and health informatics, particularly with the integration of AI in healthcare, highlighted the following key directions:

∘ *Ethical and legal concerns on e-environments in digital healthcare*—multiple ethical barriers for implementation with topics related to consent, the validation of eHealth [164], responsibility, liability, inclusiveness and diversity, the monitoring and follow-up of data output, ethical policy, guidelines and frameworks, and autonomy [165], implementation and compliance with regulations [166], ethical debates around patient autonomy and responsibility, since even though digital health tools promise greater patient empowerment through self-management, they can also place undue responsibility on individuals in terms of managing their health [167], etc.
∘ *Social challenges*—the “digital health divide”: despite the promise of digital health, underserved communities often struggle to benefit from these technologies, potentially exacerbating health outcomes [168], health surveillance, and autonomy due to the increasing use of digital health monitoring tools, such as wearables and remote tracking devices [164].
∘ *Cyber security challenges*—data breaches and privacy risks as potential risks to patient confidentiality, particularly when data are shared across platforms without strong encryption methods [169], IoMT vulnerabilities that could allow hackers to manipulate their functions, posing serious threats to patient safety [170], AI and machine learning vulnerabilities, since data poisoning attacks could be used by malicious actors to manipulate training data to produce incorrect predictions [171], etc.

## 4. Discussion

A variety of themes emerged from the articles provided by the invited experts, as well as from the discussions organised during the Symposium. Some experts focused on the historical foundations of the field to understand both the achievements and ongoing challenges, while others questioned the future pathways and the human aspects of the field.

According to many of the invited experts, the informatics in biomedicine and healthcare analysis in this work has shown that in Europe, medical informatics has been successful in combining hospital information systems with medical records data. This progress was enabled by research programming and the use of digital image processing to improve the quality of medical images. Biomedical and health informatics has also faced difficulties, including the need to educate health workers, who think it is purely a technological application, on digital literacy. The major issues involve the application of international standards, securing patient’s electronic records, addressing ethics and legal issues in telemedicine, and considering the societal factors affecting implementation. Artificial intelligence’s potential in medical care and personalised decision support needs are also part of this discussion. Additionally, interoperability, which refers to the smooth exchange of data and information between different systems, is yet another focus area within this domain.

According to the present narrative review, health data in informatics starts with digitising the healthcare processes leading to electronic health records (EHRs). EHRs are comprehensive archives with patient-specific information beyond just medical data. This digital transformation has generated vast amounts of data, creating opportunities to enhance healthcare systems. The key milestones include data collection, EHR generation, and transforming EHR data for machine understanding. Progress involves using data to improve procedures, processes, and gain insights into human health. Challenges arise with widespread EHR adoption, like data-sharing hurdles during pandemics such as COVID-19. Despite EHRs’ broad use, their full potential is not realised due to technological and acceptance barriers. Researchers are striving to advance EHR technology, recognising the need to address human-based challenges for further progress.

Many of the invited experts argued that significant progress has been made in adopting health informatics within the healthcare workforce. Various technologies have been integrated into daily practice, benefiting nurses and clinicians in patient management, care continuity, and accountability. These applications aim to enhance patient safety. Informatics also supports collaboration, cooperation, and engagement among healthcare professionals. Nurses’ involvement in technological advancements is vital for the successful implementation of information systems, especially in technologically advanced countries. The COVID-19 pandemic has accelerated the global adoption of telehealth and tele-nursing applications, leading to changes in educational methods like the increased use of gamification and simulation learning for healthcare workers.

By the 1970s, nurse organisations championed the first developments in health informatics, with Professor John Mantas from the University of Athens being a pioneer in Nursing Informatics. Nurses have contributed significantly to setting standards and clinical informatics language. The expansion of telehealth, telemonitoring, telenursing, and mobile health applications during COVID-19 marks a major milestone in health informatics.

Furthermore, this area is very active and open to new challenges in utilising health informatics by clinicians and nurses, which include fostering digital literacy, integrating information technologies into clinical practice, and embracing robotics and 3D printing in healthcare. Global interoperability solutions are crucial for managing future pandemics, while personalised health technologies will redefine patient safety.

Currently, BMHI education is closely linked to the integration of digital tools and services in healthcare. Progress varies among nations and typically involves a mix of formal and informal education. BMHI has expanded to include professions beyond medicine, such as computer scientists, lawyers, and economists. There is an increasing recognition of the importance of fostering capability and enhancing health digital literacy for all individuals.

Several invited experts advocated that BMHI education includes acknowledging the need for education and defining the discipline in academic programmes. It is essential to conduct a multidisciplinary assessment of educational needs to establish international standards for knowledge and skills. Accreditation standards are crucial in adapting to global trends and the rapid evolution of digital healthcare, undergoing regular evaluation and enhancement.

The results of the narrative review conclude that BMHI education mainly stem from the slow adaptation of traditional education systems to changes. Ensuring programme quality remains an ongoing challenge despite existing accreditation standards. The COVID-19 pandemic has introduced additional hurdles by necessitating immediate digital implementation. Particularly, innovation, evidence-based knowledge, and inclusivity need prompt attention across all age groups. Overall, the BMHI education is tied to advancements in digital tools and services in healthcare. The discipline continues to grow and extend beyond traditional boundaries, with persistent challenges requiring resolution for quality education and effective implementation.

The invited experts revealed that healthcare informatics has advanced significantly in areas like data protection and security, including the development of applications such as personal health informatics and AI-based clinical decision support systems. Studies have been conducted to ensure data and decision quality and reliability in various scenarios. Furthermore, protecting personal data and aligning digital tools with different health systems and economic statuses is crucial. While progress has been made, significant hurdles still need to be overcome.

It is worth mentioning that healthcare informatics has achieved serious progress within the fields of data protection, privacy, security, personal health informatics, and AI-based clinical decision support systems. Factors like data quality, decision accuracy, patient engagement, empowerment, and treatment performance have also been crucial. Focus areas include personal health data interpretation, data protection, aligning digital tools with health systems, and ensuring accessibility for individuals with disabilities. Furthermore, this narrative review reveals that the important parts involve addressing economic disparities, ensuring access to telecommunication channels and broadband internet, and understanding the impact of personal health informatics tools on patient engagement and empowerment. Technological innovation in healthcare informatics must also address issues like inequality, accessibility, the lack of broadband internet service in certain areas, and the limitations faced by individuals with disabilities. Developing digital health literacy is vital as exposure to digital services, especially in the health sector, continues to grow across all generations.

As mentioned in the predictions and forecasts of the medical informatics development from 2014 [5], many unexpected developments in the field of ICT have a direct influence on the healthcare system, including AI in personalised medicine, blockchain technologies for securing patient’s electronic records, etc., which was assumed as early as the beginning of 21st century [5]. The goal of “changing the world”, assigned to biomedical and health informaticians [5], was significantly accelerated during the COVID-19 pandemic, which on the other hand contributed to the changing environment that cultivated innovative projects, like shortening testing and evaluation time, fostering an increase industrial interests, as well as encouraging professionals to delve even more deeply into the work of their profession.

The research revealed numerous BMHI innovations in the field that need to be incorporated into the clinical reality. The recent pandemic has driven BMHI innovations for the future of healthcare. The research reveals that well-established but not widely used digital health tools, and other emerging digital health technologies, such as artificial intelligence, machine learning, telemedicine, telemonitoring, wearable devices, and mobile health, were boosted by the needs of the pandemic. The modern clinical needs are focused on patient-centric approaches: the enhancement of patient engagement and empowerment, as well as personalised and precision treatments. Furthermore, research reveals concerns regarding ethical and security discussions during the use of digital health technologies.

This paper exhibits particular strengths and limitations. The first strength is that the article is based on opinions of invited experts, being widely recognised as leaders and witnesses of many BMHI trends, while several of them are already citied as forecasters in education and healthcare in the information society. At the same time, the paper integrates a review of articles from the Scopus database—one of the largest sources of peer-reviewed literature—facilitating access to an extensive range of publications over a long period, including those indexed in PubMed. Another strength is a unique combination of historical overview of achieved progresses, leading to the identification of key milestones and open challenges for innovations and future development.

On the other hand, the paper also displays certain limitations, among which the most important is that the analysis was subjective as a result of the invited experts’ opinions and not based on a fully neutral literature review, using a wide definition of dimensions for critical review. For the literature review, a comprehensive search was conducted across all relevant bibliographic databases, as outlined in the Methodology section. This approach ensured a thorough examination of the available research. Future work will be focused on integrating synthetic knowledge with the presented approach.

## 5. Conclusions

This paper provides a narrative review of the progress achieved in BMHI, assesses key landmarks, and discusses future inquiries by summarising findings collected through a meticulously designed qualitative research methodology. The process began with collecting original articles from leading experts in the field, which were used for thematic analysis. This was followed by the organization of an International Symposium held in October 2022, where discussions on identified thematic dimensions took place. Finally, the knowledge generated from the articles and open discussions was synthesised and presented across five key dimensions: informatics in biomedicine and healthcare, health data in informatics, nurses in informatics, education and accreditation in health informatics, and ethical, legal, social, and security issues. Each dimension is characterised with significant advancements, accelerated mainly by the rapid development of ICTs and their comprehensive application in the field of healthcare, with the simultaneous development of BMHI as a multidisciplinary profession. A special focus was placed on discussing findings from the literature review on the most significant developments in the field, published from 2022 to the present. It provided significant insights into the potential for innovations to shape the trajectories of the future developments of digital health.

This work was shaped by insights from the invited experts, many of whom witnessed the earliest stages of this profession’s evolution. Our main goal was to create a comprehensive body of knowledge essential for future development and progress in the field. We expect this work to be vital for educators in training professionals who can tackle the challenges of designing, developing, and leading innovations. Additionally, it should assist healthcare workers in effectively integrating these innovations into their routines, ultimately enhancing health outcomes and boosting the efficiency, effectiveness, and security of the system. However, researchers and innovators are expected to benefit from a proper understanding of trends and causal effects, along with open issues and challenges, some of which date back to the early stages of BMHI.

This work could extend to new advancements involving the experts, as well as extending to areas that were not part of the Symposium. Based on the forecasters in education and healthcare in the information society, this work can be further extended to specific topics or even add new ones that arise in the future.

## Data Availability

The original dataset used in the study is openly available in Zenodo at https://zenodo.org/records/13744432 (accessed on 11 September 2024).
